# Zikavirus *pr*ME Envelope Pseudotyped Human Immunodeficiency Virus Type-1 as a Novel Tool for Glioblastoma-Directed Virotherapy

**DOI:** 10.3390/cancers12041000

**Published:** 2020-04-18

**Authors:** Maibritt Kretschmer, Patrycja Kadlubowska, Daniel Hoffmann, Birco Schwalbe, Heidi Auerswald, Michael Schreiber

**Affiliations:** 1 Department of Virology, Bernhard-Nocht Institute for Tropical Medicine, D-20359 Hamburg, Germany; 2 Department of Neurosurgery, Asklepios Klinik Nord—Heidberg, Asklepios Kliniken Hamburg GmbH, D-22417 Hamburg, Germany; 3 Virology Unit, Institut Pasteur in Cambodia, Phnom Penh 12201, Cambodia

**Keywords:** oncolytic viruses, zika virus, glioma stem cells, HIV-1, pseudotyped virus, neurotropism, HIV-1 packaging system, virotherapy

## Abstract

Glioblastoma multiforme is the most lethal type of brain tumor that is not yet curable owing to its frequent resurgence after surgery. Resistance is mainly caused by the presence of a subpopulation of tumor cells, the glioma stem cells (GSCs), which are highly resistant to radiation and chemotherapy. In 2015, Zikavirus (ZIKV)-induced microcephaly emerged in newborns, indicating that ZIKV has a specific neurotropism. Accordingly, an oncolytic tropism for infecting GSCs was demonstrated in a murine tumor model. Like other flaviviruses, ZIKV is enveloped by two proteins, *pr*M and E. The pME expression plasmid along with the HIV-1 vector pNL Luc AM generated *pr*ME pseudotyped viral particles. Four different *pr*ME envelopes, Z1 to Z4, were cloned, and the corresponding pseudotypes, Z1- to Z4-HIV*luc*, produced by this two-plasmid system, were tested for entry efficiency using Vero-B4 cells. The most efficient pseudotype, Z1-HIV*luc*, also infected glioma-derived cell lines U87 and 86HG39. The pseudotype system was then extended by using a three-plasmid system including pME-Z1, the HIV-1 packaging plasmid psPAX2, and the lentiviral vector pLenti-luciferase-P2A-Neo. The corresponding pseudotype, designated Z1-LENTI*luc*, also infected U87 and 86HG39 cells. Altogether, a pseudotyped virus especially targeting glioma-derived cells might be a promising candidate for a prospective glioblastoma-directed virotherapy.

## 1. Introduction

Glioblastoma multiforme (GBM), the most common and most aggressive version of primary brain tumors, carries a really dismal prognosis [1,2]. Self-renewal and resistance to conventional therapy are the main features of malignant glioma and the location of the tumors in the most complex organ, the brain, is another demanding challenge for an effective tumor therapy. Neural tumor precursor cells, the so-called glioblastoma stem cells (GSCs), have been identified as the cell population, which owns an explicit resistance to radiotherapy and chemotherapy [3]. Thus, these cells are believed to be the driving force for the recurrence of the disease and, therefore, GSCs are a preferred target for therapeutic concepts [4]. A modicum of hope has become visible by the results of experimental studies and clinical trials using viruses with a tropism for glioma cells and GSCs. Historically, oncolytic virotherapy is a concept with a long history [5] that was first described back in 1912, when regression of cervical cancer was seen owing to the administration of the Louis Pasteur rabies vaccine [6]. In the last decades, the understanding of virus and tumor genetics has improved as a result of improved molecular biology, leading to the generation of recombinant, genetically modified virus candidates. Clinical trials have been completed using modified versions of herpes simplex virus [7,8,9,10,11,12], adenovirus [13,14], Newcastle disease virus [15], reovirus [16,17], parvovirus [18], and poliovirus [19]. Some of these studies demonstrated that a small subset of patients had a benefit from such virotherapy applications, but the statistical significance of these findings must be demonstrated in larger control trials [20].

A new discovery, with a high impact on the future aspects of glioma virotherapy, was that Zikavirus (ZIKV) showed a specific neurotropism for glial cells [21]. ZIKV is a new emerging mosquito-borne virus belonging to the *Flaviviridae* family [22]. In the *Flaviviridae* family, all members are enveloped viruses with two external, membranous proteins, the envelope E and the precursor of the membrane protein *pr*M [23], both relevant for viral entry. In early 2015, abnormal rates of ZIKV infections were seen in Brazil, and in late 2015, an unexpected high number of newborns showed microcephaly, a disease where a baby’s head is much smaller than expected. Altogether, the new emerging ZIKV was shown to be the cause of Guillain–Barré syndrome (GBS), microcephaly, and other congenital brain abnormalities, proving its neurotropic phenotype [24]. An interesting finding was the oncolytic activity of ZIKV against GSCs [25]. A selective effect of ZIKV was observed for GSCs, causing loss of self-renewal and proliferation in glioblastoma organoids. In mice, ZIKV infection prolonged survival by slowing down tumor growth and showed only a weak side effect on normal neural cells [25]. Following these observations, the safety and efficiency of a live attenuated ZIKV candidate was investigated [26]. In an orthotopic glioma model, the live attenuated ZIKV reduced intracerebral tumor growth and prolonged animal survival. In these studies, a selective killing on GSCs within the tumor was observed [26]. Recent findings demonstrate that the entry of ZIKV into GSCs is supported by Integrin α_v_β_5_ because viral entry can be specifically blocked by an α_v_β_5_-specific antibody or by the cyclic peptide Cilengitide (cyclo-RGDfV-) [27], a peptide that is highly specific for binding to α_v_ integrin [28,29,30]. Integrin α_v_ as part of the integrins α_v_β_3_ and α_v_β_5_ have been shown to play a role in a variety of tumors, particularly malignant gliomas. In high-grade gliomas, the expression level of α_v_β_3_ is significantly higher not only in endothelial, but also in tumor cells compared with low-grade gliomas [31]. Additionally, α_v_ as part of α_v_β_5_ occurs frequently on cells from highly malignant gliomas [32]. Thus, the molecules used by ZIKV for viral entry are predominantly expressed in tumors, but can hardly be found in the tissue of the normal brain [33]. After ZIKV entry via its integrin receptors, the upregulation of micro-RNA-34c was observed in infected GSCs, suggested to be the reason for the reduction of GSC growth in an in vitro cell culture model using GSC spheres [34].

The ZIKV is enveloped by the two proteins *pr*M and E. Proteolytic cleavage of *pr*M by the host cell protease furin causes the transformation of the non-infectious *pr*ME complex into the infectious ME-envelope. Viral entry is achieved by binding of the protein E to its receptors via its third domain followed by the membrane fusion process, initialized by the tip of the E protein called the fusion loop [35,36]. As explained above, the ZIKV possesses the characteristic to infect and to kill GSCs [25]. Therefore, the ZIKV *pr*ME envelopes are the preferred structures that should be used to construct a virus particle as a new candidate for virotherapy. All the ZIKV approaches that showed an oncolytic effect is utilizing a wild-type virus, but such an injection of active replicating ZIKV into the brain, even when attenuated, raises safety concerns. Our approach, therefore, was to combine the ZIKV specificity for glioma cells located in the *pr*ME envelope with the viral vector technology for the generation of lentiviral pseudotypes. Such pseudotypes are infectious, but replication incompetent particles, allowing only viral entry into cells—a process described as single-round infection. Another aspect is the use of the viral vector as a Trojan horse for the delivery of genetic markers or anti-tumor active genes into the target cell.

In the present study, we have shown for the first time the successful generation of an infectious flavivirus pseudotype using the HIV packaging system, a combination of ZIKV *pr*ME as the viral envelope and HIV-1 as the core structure. The pseudotype infected the standard ZIKV host cells Vero-B4 and, most importantly, owned the capacity to infect glioma-derived cell lines, demonstrated by infection of U87 and 86HG39 cells.

## 2. Results

### 2.1. Zikavirus prME Pseudotyped HIV-1

As shown in Table 1, four different pseudotypes were synthesized, designated Z1-, Z2-, Z3-, and Z4-HIV*luc*. Pseudotypes were generated by transfection of HEK293T cells with one of the *pr*ME expression plasmids together with the pNL Luc AM plasmid.

**Figure 1:**
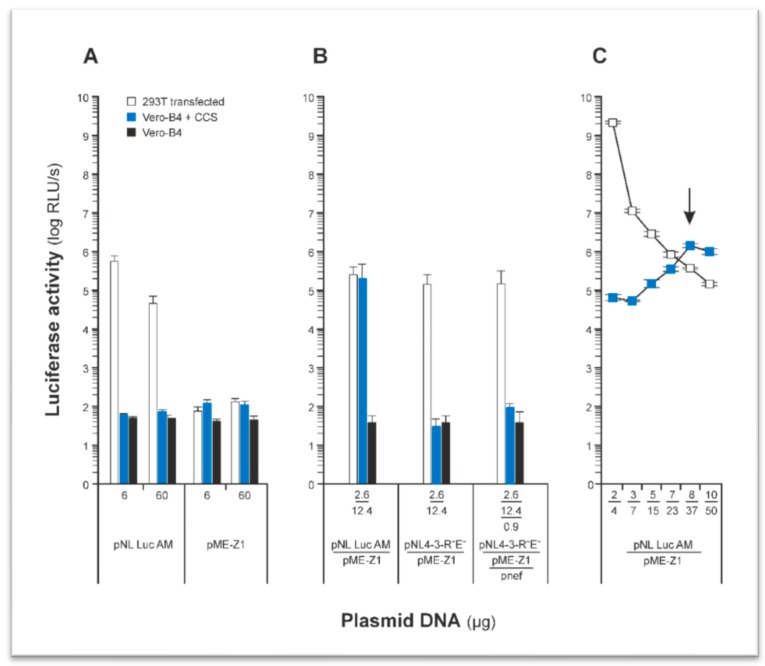
Luciferase activity in transfected HEK293T cells and Z1-HIV*luc* infected Vero-B4 cells transfected with different amounts of plasmids. (**A**) Transfection and infection controls. (**B**) Comparison of pseudotype production by viral vectors pNL Luc AM (*nef*^+^) and pNL4-3-R^−^E^−^, (*nef*^−^). pnef, plasmid for expression of the HIV-1 *nef* protein. pME-Z1, expression of *pr*ME. Transfections for the comparison were carried out in 24 wells, in contrast to experiments described in (**A**) and (**C**). Cell culture supernatants (CCSs) were tested at a 1:5 dilution for infection of Vero-B4 cells. Numbers indicate µg of plasmid DNA used for transfection. (**C**) From HEK293T transfected cells, CCS was diluted 1:5 and used for infection of Vero-B4 cells. Plasmid ratios (in µg) of pNL Luc AM and pME-Z1 were as follows: 6 = 2 + 4, 10 = 3 + 7, 20 = 5 + 15, 30 = 7 + 23, 45 = 8 + 37, and 60 = 10 + 50. pNL Luc AM: vector for HIV-1 core production (*env*^−^, *luc*^+^, *nef*^+^). pME-Z1: Zika *pr*ME envelope expression plasmid. Y-axis: relative light units (RLU) measured in HEK293T cells transfected with plasmids (white squares or bars) or (**A**) and (**B**) Vero-B4 cells CCS inoculated (blue bars) or (**C**) infected with Z1-HIV*luc* containing CCS (blue squares). Black bars: luciferase background activity in non-transfected and non-infected Vero-B4 cells. X-axis: µg plasmid DNA used for transfection of HEK293T cells.

First of all, the transfection protocol for pseudotype production was optimized. As shown in Figure 1, the highest rate for infectious particles in HEK293T cell culture supernatants (CCSs) was observed using 8 µg pME-Z1 and 37 µg pNL Luc AM plasmid DNA (Figure 1C, black arrow). Lower DNA amounts showed higher luciferase expression in the transfected HEK293T cells (Figure 1C, white symbols), but in the corresponding CCSs, the titers of infectious particles were lower. Thus, for all other experiments, we used the amount of 8 + 37 µg plasmid DNA for transfection in the six-well format. Cell culture supernatants showed a p24 antigen equivalent of 5 ± 0.8 pg/mL for Z1-HIV*luc*.

Using the combination of Z1-HIV*luc* and pNL Luc AM, we showed that a functional and infectious pseudotype was generated that had the capacity to infect cells from the Vero lineage, the standard cells for flavivirus propagation.

### 2.2. Efficiency of Zika prME Pseudotypes for Infection of Vero-B4 Cells

**Figure 2:**
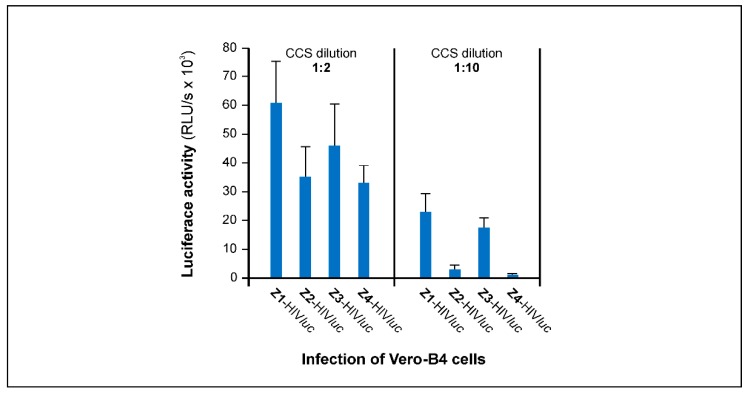
Efficiency of Vero-B4 infection by Zika *pr*ME pseudotypes. CCSs containing the Z1 to Z4-HIV*luc* pseudotype particles were tested at 1:2 and 1:10 dilution. Luciferase activity was monitored on day 3 post infection. Dilutions 1:2 represent 0.25 pg p24/96-well. The RLU/s data are means of triplicate infections using a CCS from one transfection.

All four pseudotypes, Z1-, Z2-, Z3-, and Z4-HIV*luc*, were tested for infection of Vero-B4 cells. Therefore, HEK293T cells grown in six wells were transfected with 8 µg pNL Luc AM and 37 µg pME-Z1-to-Z4 plasmids, respectively. Cell culture supernatants (CCSs) were harvested on day 3 and were diluted 1:2 and 1:10 in DMEM medium. Vero-B4 cells were cultured in 96 wells and 100 µL of the CCS dilutions was added to each of the wells. All infection experiments were performed in triplicate and luciferase activity in Vero-B4 cells was analyzed three days post infection. As shown in Figure 2, the most efficient pseudotype for Vero-B4 infection was Z1-HIV*luc* with 60.5 10^3^ relative light units (RLU)/s followed by Z3-HIV*luc* with 45.9 × 10^3^ RLU/s, and Z2-HIV*luc* and Z4-HIV*luc* with 34.9 × 10^3^ and 32.9 × 10^3^ RLU/s, respectively (CCS dilutions 1:2). Z1-HIV*luc* and Z3-HIV*luc* also showed the highest infection values at 1:10 dilution with 22.8 × 10^3^ and 17.4 × 10^3^ RLU/s, respectively. As Z1-HIV*luc* had the highest RLU values in both tests, we used the pME-Z1 expression plasmid in all other experiments to produce Z1-HIV*luc* and Z1-LENTI*luc* pseudotypes.

### 2.3. Infection of U87 and 86HG39 Cells by Z1-HIV-luc

The Z1-HIV*luc* pseudotype generated by the two-plasmid system (Appendix A, Figure A2) was used to test glioblastoma-derived laboratory cell lines U87 and 86HG39 for pseudotype particle permissiveness. HEK293T cells were grown in 24 wells and transfected with 2.4 µg pNL Luc AM and 12.6 µg pME-Z1 plasmids. Cell culture supernatants were harvested on day 3 and used for the infection of Vero-B4, 86HG39, and U87 cells at various dilutions (1:4–1:4096, Figure 3). Luciferase activity in the infected cells was analyzed three days post infection. All infections were carried out in quadruple and the standard deviation of the means was ≤20%. The highest RLU values were obtained for Vero-B4 infection with 8.3 × 10^4^ RLU/s, followed by infection of 86HG39 with 2.7 × 10^4^ and U87 with 7.0 × 10^3^ RLU/s at a 1:4 dilution. The controls (CCS from pNL Luc AM transfected HEK293T cells) were all in a range between 58 and 32 RLU/s, thus the infections measured for the three cell lines were clearly above the cutoff level (180 RLU/s) of the infection assay.

### 2.4. Infection of Cells by the Z1-LENTIluc Pseudotype Synthesized Using a Three-Plasmid System

**Figure 4:**
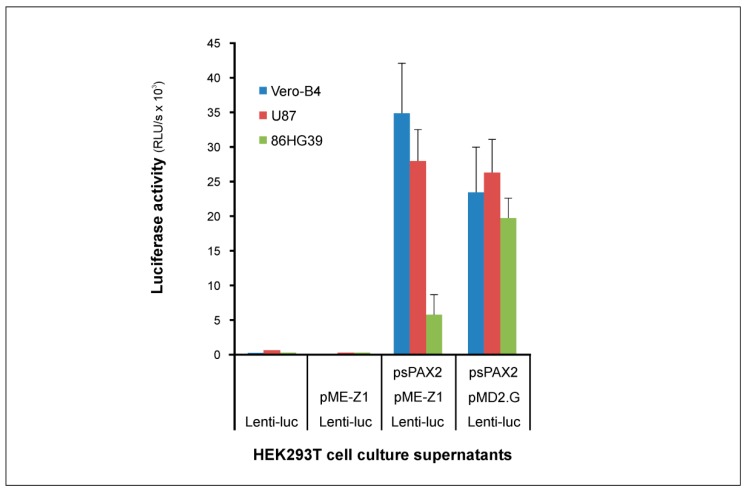
Infection of cells by Z1-LENTI*luc.* The pseudotype Z1-LENTI*luc* was synthesized using the three plasmids pME-Z1, psPAX2, and pLenti-luciferase-P2A-Neo (Lenti-luc). Cell culture supernatants from HEK293T transfected cells were tested at 1:5 dilutions on cell layers grown in 96-well cell culture plates. Luciferase activity in target cells was measured at day 3 post infection. Pseudotypes with VSV-G envelope expressed by pMD2.G were used for comparison.

As ZIKV *pr*ME pseudotypes synthesized by the two-plasmid system showed infection of glioma cell lines, we wanted to investigate infectivity of pseudotypes produced by a three-plasmid lentiviral system. The advantage of a three-plasmid system is that the freed space in the *gagpol* deleted viral genome can be used for any kind of gene delivery into permissive cells. The three-plasmid system used for the generation of LENTI*luc* pseudotypes contains (i) the pME-Z1 plasmid for the expression of the ZIKV envelope proteins *pr*M and E, (ii) the so-called packaging plasmid psPAX2 for the expression of the HIV-1 precursor’s *gag* and *gagpol*, and (iii) the pLenti-luciferase-P2A-Neo plasmid coding for HIV-1 genomic RNA with the relevant elements for genome replication and the reporter gene for the expression of the firefly luciferase. For generation of the Z1-LENTI*luc* pseudotype, HEK293T cells grown in 24 wells were transfected with 2.6 µg pLenti-luciferase-P2A-Neo, 12.4 µg pME-Z1 (Zika *pr*ME) or pMD2.G (VSV-G), and 5 µg psPAX2 plasmid DNA. Cell culture supernatants were harvested on day 3 and used at a 1:5 dilution for infection of cells grown in 96 wells. All infections were carried out in triplicate with a standard deviation of the means ≤20%. As shown in Figure 4, the highest infection values were observed for Vero-B4 with 3.5 × 10^4^ RLU/s. Infection of U87 cells was similar to Vero-B4 with 2.8 × 10^4^ RLU/s. Infection of 86HG39 cells was significantly lower with 5.8 × 10^3^ RLU/s, but RLU values were clearly above the luciferase background of the infection assay. For comparison of the infection rates, cells were infected with pseudotype particles containing the VSV-G envelope, indicating that all three cell lines can be infected at similar rates by the VSV-G pseudotype and that Z1-LENTI*luc* infection is comparable to the VSV-G infection, with the exception of the Z1-LENTI*luc*/86HG39 combination. Z1-LENTI*luc* showed low infection rate on 86HG39 cells in contrast to the Z1-HIV*luc* particles produced by the two-plasmid system. For the generation of Z1-LENTI*luc* using the three-plasmid system, we used the most widely used packaging plasmid psPAX2. This plasmid expresses HIV-1 *gag*, *gagpol*, *rev*, and *tat*, but is *nef* deficient in contrast to pNL Luc AM, which is *nef* positive. This might explain the differences between Z1-HIV*luc* and Z1-LENTI*luc* infections of 86HG39 cells.

## 3. Discussion

The concept of oncolytic virotherapy generally uses replication competent viruses to infect cancer cells with the aim to destroy them. Cell death will be induced owing to direct virus-induced cytotoxicity or indirect cytotoxic immune effector mechanisms [37]. Additionally, oncolytic viruses can be used to inhibit tumor growth by preventing angiogenesis, or through the expression of proteins triggering an anti-cancer immune response [38,39,40,41]. In general, each virus has a specific cellular tropism that determines which cells will become infected. For example, HIV-1 uses the CD4 receptor on T-helper cells, damaging the immune system in the most negative way, and finally leading to AIDS. However, the virus specificity for CD4 can be used in a positive way, which is the CRISPR/Cas9 knockdown of the CCR5 and CXCR4 co-receptors in CD4+ T-helper cells by a genetically engineered, replication incompetent retrovirus. Thus, autologous T-helper cells become resistant against HIV-1 [42]. Although such an application is restricted to the in vitro manipulation of T-helper cells, these experiments demonstrate that engineered retroviruses are excellent gene delivery systems and, therefore, provide important tools for therapeutic anti-tumor strategies [43].

With the emergence of the new neurotropic ZIKV with its unexpected preference for infecting GSCs, a new research area for glioma virotherapy opened up [26,28,29,30,43,44]. In regard to *Flaviviridae*, recombinant reporter viruses, for example, for West Nile and Dengue virus, were constructed using the original flavivirus genome as a packaging template [45,46]. Moreover, pseudotypes between yellow fever and West Nile viruses were constructed to study antibody responses against yellow fever [47]. In addition, a pseudotype system for flaviviruses was developed based on a *pr*ME deficient Dengue-1 genome [48]. This system allows the study of neutralizing antibodies as it produces single-round infectious particles carrying a luciferase reporter gene. All of these concepts use the flavivirus RNA genome as a template for the production of the pseudotype core, and thus have their restrictions as gene delivery tools.

For the production of envelope-pseudotyped viruses, the murine leukemia virus (MLV), the vesicular stomatitis virus (VSV), and the lentiviral vector packaging systems are used mainly because of their high efficiency and easy handling. The easiest way to get an envelope-pseudotype is the co-expression of the envelope together with an envelope deleted (*env*^−^) viral vector, the so-called 2-plasmid system. An overview of the current status of envelope-pseudotype systems was given by Li et al., 2018 [49]. Using the two-plasmid, co-transfection system was sufficient for many different virus envelopes, but had not been conducted successfully for *Flaviviridae* including ZIKV [49]. Thus, ZIKV envelope-pseudotypes using the HIV-1 lentiviral packaging system, as described in our study, are new and not described in the literature up to now. In a first experiment, we used the HIV-1 viral packaging vector pNL4-3-R^−^E^−^, which works very effectively for *env*-pseudotypes of HIV [50], Ebola [51], SARS-CoV [52], MERS-CoV [53], and influenza [54,55]. Unfortunately, this vector was highly insufficient in *pr*ME-pseudotype production. As the vector was *nef*-depleted, we changed our strategy by using the *nef*+ viral vector pNL Luc AM, inspired by a publication describing a new function of HIV-1 *nef* [56], that is, its impact on cytoplasmic delivery of the HIV-1 genome. Using the *nef*+ vector, significant amounts of pseudotype particles were detected in cell culture supernatants. Interestingly, when the transfection of cells was optimized for cellular luciferase activity, the titers of infectious pseudotype particles were low. In contrast, pseudotype titers increased significantly when the amounts of plasmid DNA for transfection were enhanced. Thus, the highest titers for pseudotype particles were observed when luciferase activity in the transfected cells was low. This observation was a breakthrough in the production of the ZIKV *pr*ME-pseudotypes, allowing further pseudotype infection experiments under controlled and reproducible conditions.

The U87 cell line (formerly known as Uppsala 87 Malignant Glioma) [57] and the 86HG39 cell line [58] are human primary glioblastoma cell lines commonly used in glioma research. We used these cell lines to investigate if they were permissive for the ZIKV *pr*ME-pseudotypes. Compared with Vero-B4 cells, both glioma cell lines were efficiently infected by Z1-HIV*luc*. Vero cells were not used with the primary focus if their integrin expression was similar to glioma-derived cell lines. They are used as an infection control for the pseudotypes as these cells are widely used for isolation and cultivation of several viruses, including flaviviruses. Analysis of Vero cells revealed the expression of several integrins, especially α_v_β_3_, but their expression level in comparison with glioblastoma cells is not known [59,60]. The infection experiments can be interpreted as a proof-of-principle: co-transfection of the *pr*ME expression vector together with pNL Luc AM (*nef*+) produced functional pseudotype particles and these particles showed infectivity for glioma cell lines at similar rates compared with Vero-B4 cells. Thus, the Z1-HIV*luc* pseudotype is a new tool to study the permissiveness of glioma cell lines as well as primary tumor cells as the next step.

On the basis of these findings, we also generated the *pr*ME-pseudotype Z1-LENTI*luc* using a three-plasmid system. The pseudotype core structure was produced by the HIV-1 precursor proteins *gag* and *gagpol* now expressed by the third packaging plasmid psPAX2. We used psPAX2 for packaging as it is the most widely used plasmid for generation of HIV-based pseudotypes. The lentiviral vector used in our application carries no *gag*, *pol*, or *env* genes and the freed space can now be principally used for a large variety of gene applications. We used a lentiviral vector that expressed the genes for firefly luciferase, as a reporter, and the gene for neomycin resistance. Together with the pME-Z1 plasmid, infectious particles were obtained and infection of Vero-B4, U87, and 86HG39 cells was observed. Using the three-plasmid system, infection rates were generally lower because three plasmids instead of two plasmids have to find their way into the target cells. Interestingly, infection of 86HG39 cells with Z1-LENTI*luc* was lower compared with Z1-HIV*luc* or U87 cells. This might be because psPAX2 is *rev*+ and *tat*+, but lacks the expression of the *nef* gene. The difference in infectivity of Z1-LENTI*luc* also indicates that *nef* might play a role in viral entry related to the origin of the glioma cell lines. Probably because receptor binding and membrane fusion is started by ZIKV protein E, but most importantly, HIV-1 capsid cytoplasmic delivery is, in some cases, *nef* dependent [56]. However, using the three-plasmid system, the lentiviral vector was transmitted successfully into glioma cells. An optimized packaging system for Z1-LENTI*luc* would probably be a single plasmid for the expression of *gag*, *gagpol*, *rev*, *tat*, *nef*, all together with *pr*ME. Another option would be to establish a stable cell line expressing all these proteins necessary for particle formation.

For lentiviral vectors based on the HIV-1 genome, a large variety exists including vectors with luminescence or fluorescence reporter genes, operons for the expression of foreign genes including anti-tumor-genes, or vectors transmitting the complete CRISPR/Cas9 system [61,62]. Thus, using these kinds of lentiviral vectors and packaging systems together with the ZIKV *pr*ME-dependent neurotropism paves the way for new viral particles specifically targeting glioma cells. The high infectivity of the Z1-HIV*luc* pseudotype, generated by the two-plasmid system, is the key for future experiments, in order to study infectivity of the Z1-HIV*luc* pseudotype for cells directly isolated from brain tumors.

## 4. Materials and Methods

### 4.1. Plasmids

**Figure A1:**
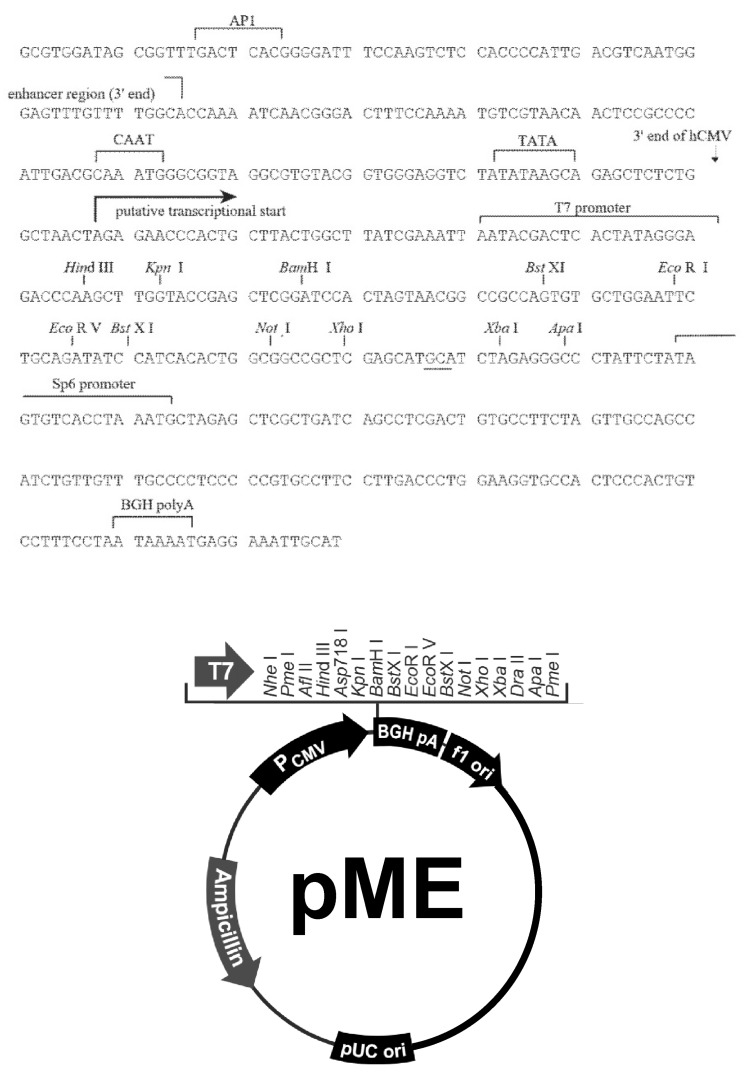
Map of the pME expression plasmid. PCMV, promotor of human cytomegalovirus; BGH pA, bovine growth hormone poly A signal; ori, origin of replication from pUC or bacteriophage f1; Ampicillin, gene for Ampicillin resistance ampR.

**Figure A2:**
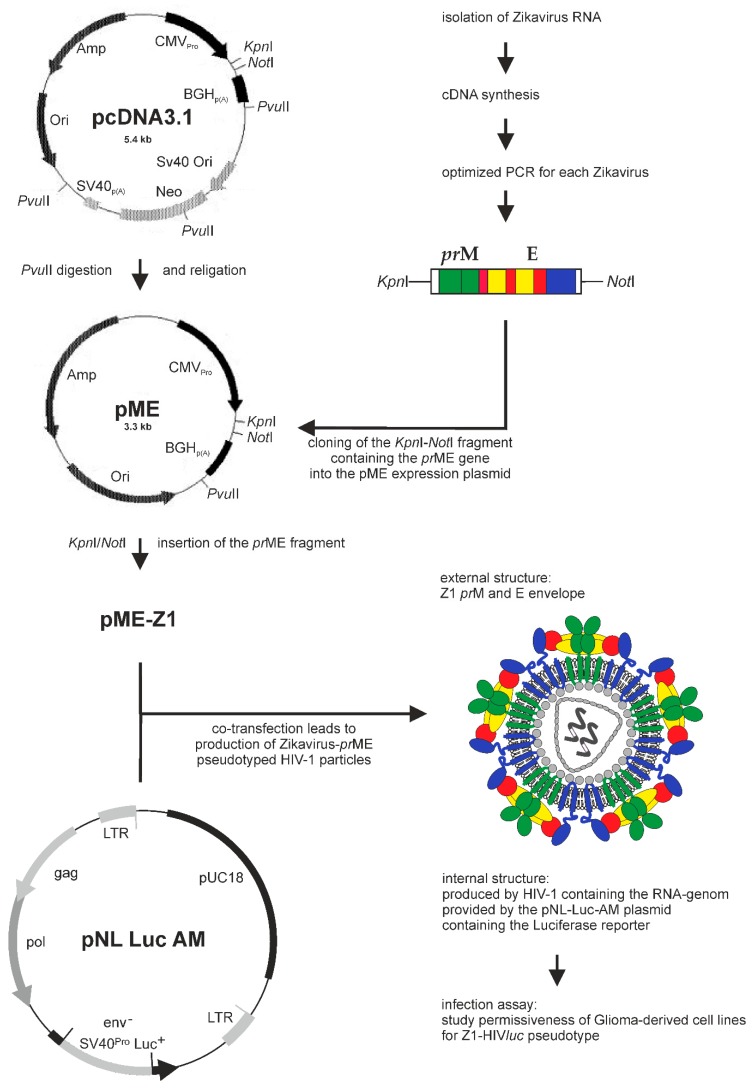
Schematic diagram of the cloning procedure for the production of Zika-HIV*luc* pseudotypes. LTR, long terminal repeat.

Plasmid pME, a modified version of pcDNA3.1 (invitrogen), lacks the *Pvu*II-*Pvu*II fragment containing the SV40_ORI_ and SV40_PA_ elements (Figure A1 and Figure A2, Bernhard-Nocht Institute for Tropical Medicine, Hamburg, Germany). The map of the plasmid is shown in Appendix A as Figure A1. Plasmids pNL4-3-Luc-R^−^E^−^, an HIV-1 NL4-3 luciferase reporter vector that contains defective *nef*, *env*, and *vpr* [63] and pnef, were a gift from H. Schaal, Heinrich-Heine University. pNL Luc AM, an HIV-1 NL4-3-based luciferase reporter vector that contains the SV40pro controlled firefly luciferase gene that replaced the HIV-1 *env* (Figure A2). This plasmid was a gift from A. Trkola, University of Zurich, and J.P. Moore, Cornell University [64]. pLenti-luciferase-P2A-Neo was a gift from Christopher Vakoc (addgene plasmid #105621) [65]. psPAX2, the HIV packaging plasmid (addgene plasmid #12260) and pMD2.G, the expression vector for VSV-G (addgene plasmid 12259), were a gift from Didier Trono.

### 4.2. Cells

HEK293T cells (CRL-11268) were from Friedrich Löffler Institute, Riems-Greifswald, Germany. U87 cells from Dr. Bruce Chesebro were obtained through the NIH AIDS Reagent Program, Division of AIDS, NIAID, NIH [66]. Vero-B4 (ACC-33) cells were from Deutsche Sammlung von Mikroorganismen und Zellkulturen, Braunschweig, Germany. 86HG39 (CVCL_7259) cells were kindly provided by T. Jacobs, Bernhard Nocht Institute for Tropical Medicine, Hamburg, Germany.

### 4.3. Zikavirus RNA

For cloning of ZIKV *pr*ME-pseudotypes, we used ZIKV RNAs from Dakar41519 (GenBank HQ234501) designated Z1, Dakar41524 (GenBank KX601166) designated Z2, and ArD158084 (GenBank KF38119) designated Z3, with all three provided by Institute Pasteur Cambodia, and H/PF/2013 (GenBank KJ776791) from European Virus Archive goes global (EVAg), Unit des Virus Emergents, Marseille, France, designated Z4. A summary of the ZIKVs used is also shown in Table 1.

### 4.4. Oligonucleotides

Oligonucleotides for cDNA synthesis were from Thermo Fisher Scientific as part of the commercial cDNA synthesis kit (RevertAid H Minus First Strand cDNA Synthesis Kits, Thermo Fisher Scientific, Dreieich, Germany). Oligonucleotides for PCR amplification were synthesized by metabion (Planegg/Steinkirchen, Germany) and adjusted in H_2_O to a final concentration of 10 pmol/µL. Oligonucleotides used for the synthesis of the four ZIKV pseudotypes Z1-to-Z4-HIV*luc*:

- Z1-Z3: *Kpn*I-for: 5′-CTT GGT ACC GCC GCC GCC ATG GGC GCA GAC ACC AGC ATC GG-3′
- Z1-Z3: *Not*I-rev: 5′-CTT CGA GCG GCC GCT CAA CTA ATT AAG CAG AAA CAG CCG TGG-3′
- Z4: *Kpn*I-for: 5′-CTT GGT ACC GCC GCC GCC ATG GGC GCA GAT ACT AGT GTC GG-3′
- Z4: *Not*I-rev: 5′-CTT CGA GCG GCC GCT CAA CTA ATT AAG CAG AGA CAG CTG TGG-3′

### 4.5. DNA Sequencing

The pCR-2.1 subcloning and pME expression plasmids were sequenced by a commercial sequencing service, LGC Genomics GmbH (Berlin, Germany), using the M13(-21)F and M13(-29)R for pCR-2.1 and the T7prom and pcDNA3.1R primers for the pME plasmids. All primers were provided by LGC Genomics.

### 4.6. Cloning of Zikavirus Envelopes

For cloning of ZIKV *pr*M and E envelope proteins, we used the provided RNAs, as shown above, as templates for reverse transcription. The cDNA synthesis was carried out using a commercial kit (RevertAid H Minus First Strand cDNA Synthesis Kits, Thermo Fisher Scientific, Germany) by applying 5 µL of the provided RNA as a template for reverse transcription in a total volume of 20 µL. For amplification, 1 µL of the cDNA mixture was given to 31.5 µL H_2_O, 5 µL PCR buffer (200 mM Tris-HCl pH 8.4, 500 mM KCl), 5 µL 25 mM MgCl_2_, 1 µL 10 mM dNTP, 2.5 µL *Kpn*I-for primer (10 pmol/µL), 2.5 µL *Not*I-rev primer (10 pmol/µL), and 1.5 µL *Taq*-DNA-polymerase (Thermo Fisher Scientific, Germany). The mixture was cycled 35 times at 95 °C (30 s), 61 °C (30 s), and 72 °C (120 s) in a Mastercycler gradient (Eppendorf AG, Hamburg, Germany). The PCR product was purified by agarose gel electrophoresis using the QIAquick Gel Extraction Kit (QIAGEN GmbH, Hilden, Germany) according to the manufacturer’s recommendations. Plasmid DNA was eluted from the column by two elution steps into a final volume of 50 µL of H_2_O. Purified DNA was cloned into the pCR-2.1 plasmid and clones were analyzed for correct M and E sequences. Positive candidates were used to cut out the ME-*Kpn*I-*Not*I fragment that was purified by agarose gel electrophoresis, as described above. The purified ME-*Kpn*I-*Not*I fragment was finally cloned into the *Kpn*I and *Not*I digested pME expression plasmid. A schematic diagram of the ME cloning procedure is shown in Appendix A as Figure A2.

### 4.7. Plasmid DNA for Transfection

All plasmids were purified by the classical CsCl large scale isolation technique described by Maniatis et al. [67]. Purified plasmid DNA was adjusted to 1 µg per µL using a UV/VIS spectral photometer (Shimadzu UV160A) using the super micro cell holder (Kyoto, Japan). For cloning of ZIKV M and E envelope proteins, we used the provided RNAs (Table 1) as templates.

### 4.8. Pseudotype Particle Production

Pseudotyped viral particles (ZIKV *pr*ME-HIV*luc*) were generated by transfection of HEK293T cells using plasmid pNL Luc AM and pME-Z1. Transfection was carried out according to the manufacturer’s recommendations (ScreenFect-A, Incella, Eggenstein-Leopoldshafen, Germany). In general, HEK293T cells were grown in six-well plates (TPP, no. 92006, Trasadingen, Switzerland) to 70–80% confluence. For a transfection, a total amount of 45 µg of plasmid DNA, 8 µg of pNL Luc AM, and 37 µg of pME-Z1 plasmid DNA was mixed with 75 µL dilution buffer (ScreenFect-A, Incella). The mixture was incubated for 5 min at room temperature and then added drop-wise to the ScreenFect-A reagent solution (6 µL reagent and 120 µL dilution buffer) and incubated for 20 min at room temperature. In the meantime, the cell culture medium was replaced and 1250 µL of fresh DMEM medium (10% FCS, 2mM L-Alanyl-L-gluatmine) was added to each well. The plasmid solution was added and the cell culture plates were incubated for 3 h at 37 °C. After transfection, the medium was replaced against 3 mL of fresh DMEM. Cell culture supernatants were harvested on day 3 after transfection, stored for 3–7 days at 8 °C, or stored in aliquots at −70 °C.

### 4.9. p24-Assay

For p24 detection in cell culture supernatants of HEK293T transfected cells, an in house p24-ELISA was used. In brief, ELISA plates (Nunc, maxisorb 96-well) were coated with 100 µL/well of anti-HIV-1-p24 D7320 (10 µg/mL; Aalto Bio Reagents, Dublin, Ireland). After blocking with PBSTM (PBS; 0.1% Tween 20; 3% low-fat milk) the plates were washed with PBST and incubated with 100 µL of the test solution for 1 h at room temperature. Plates were washed with PBST and bound p24 was detected using a polyclonal anti-p24 rabbit serum (kindly provided by H.-G. Kräusslich*,* Universitätsklinikum Heidelberg, Germany). Alternatively, the BC 1071 monoclonal anti-HIV-1-p24 antibody (Aalto Bio Reagents, Ireland) can be used for p24 antigen detection. Bound anti-p24 antibodies were detected using an anti-rabbit IgG antibody conjugated to horseradish peroxidase (BioRad, Feldkirchen, Germany). Plates were washed with PBST and PBS and 50 µL of TBM (3,3′,5,5′-tetramethylbenzidine) substrate solution was added (SureBlue TBM substrate, medac, Wedel, Germany). After incubation for 15 min, 50 µL of TMB-Bluestop solution (medac, Wedel, Germany) was added. Density of the blue staining was measured at 620 nm.

### 4.10. Infection of Vero-B4, U87, and 86HG39 Cells

The infection of cells with cell culture supernatants containing ZIKV ME pseudotyped HIV-1 particles was performed in white, for cell culture treated (Nuclon Delta Surface) 96-well plates (Thermo Fisher Scientific, Cat. No. 136102). Prior to infection, cells were seeded into the wells in DMEM (10% FCS, 2mM L-Alanyl-L-gluatmine) to reach 70–80% confluence on the next day. Incubation was carried out in a CO_2_ incubator (BBD 6220, Heraeus, Germany) at 37 °C and 5% CO_2_. For infections, cell culture supernatants were discarded and DMEM medium together with the pseudotype-containing samples at a final volume of 100 µL was added into each well. For infection, the 96-well plates were incubated for 1 h (Heraeus, BBD 6220; 37 °C, 5% CO_2_). After 1 h, 100 µL medium was added into each well and the plates were incubated for at least two days before the infection efficiency was measured by the luciferase reporter assay.

### 4.11. Luciferase Reporter Assay

The remaining HEK293T cells from the Z1- to Z4-HIVluc production step were transferred into 1.5 mL reaction tubes, centrifuged for 30 s at 10,000 rpm, and suspended in 100 µL lysis buffer (1% Triton X-100, 10% Glycerol, 25 mM Tris pH 7.5, 2 mM EDTA, 2 mM DTT). Cell lysis was carried out for 10 min at room temperature (RT) and samples were stored at 20 °C. To measure their luciferase activity, 10 µL of the cell lysate and 40 µL water were given into a well of a white polystyrene 96-well plate (non-treated surface, flat white bottom, Nunc #236105) and were mixed with 50 µL luciferase assay reagent (Luciferase Assay System, Promega #E1483, Walldorf, Germany). Plates were incubated for 2 min and measured using a 96-well plate reader (luminometer Centro LB960, Berthold Technologies, Bad Wildbad, Germany) programmed for (i) shake by plate for 2 s (speed low, diameter 0.1) and (ii) measurement by well for 1 s. The luciferase activity in the Z1-HIVluc infected adherent cell layer was measured directly in 96-well plates. Therefore, the clear bottom of the white 96-well plates was sealed with white vinyl tape (Nunc # 236272). Adherent cells were washed using PBS and, to each of the empty wells, 100 µL of PBS and 100 µL of luciferase assay reagent (Bright-Glo™ Luciferase Assay System, Promega) were added. The plates were incubated for 5 min at room temperature and the luciferase activity was measured as described above using a Centro LB960 luminometer.

## 5. Conclusions

The neurotropism of ZIKV for GBCs was used to generate HIV-1 particles that carry the ZIKV *pr*M and E envelope on the surface of the so-called pseudotyped virus. These pseudotypes are able to invade Glioblastoma derived cell lines. As the viral genome can be used for expression of reporter genes, the CRISPR/Cas9 system as well as other anti-tumor factors these ZIKV *pr*ME-pseudotypes of HIV-1 are a new tool for gene transfers and manipulations of glioma cells. Besides the importance as an anti-tumor tool, pseudotyped HIV-1 containing flavivirus *pr*M and E proteins, and especially ZIKV *pr*M and E, are completely new constructs and, until today, are not described in the literature.

## Figures and Tables

**Figure 3:**
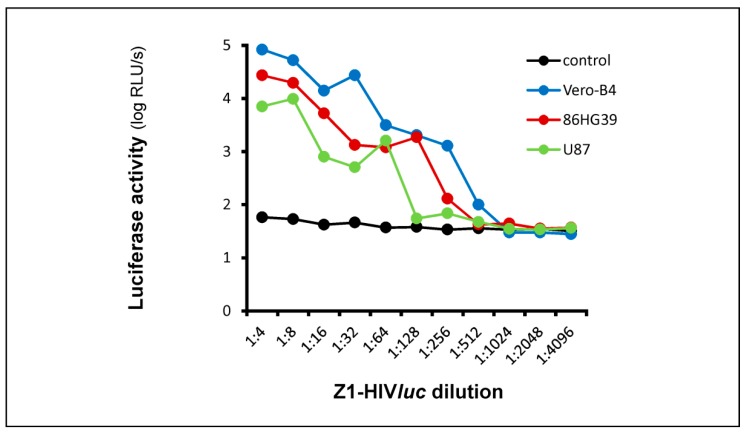
Luciferase activity in Z1-HIV*luc* infected cell lines. CCS containing Z1-HIV*luc* was tested for infection of Vero-B4, 86HG39, and U87 cells at various dilutions. CCS from pNL Luc AM transfected HEK293T cells was used as a negative control. All RLU/s data are means of quadruple infections using a CCS from one transfection. For all data points, the standard deviation of the means was ≤20% (controls ≤12%).

**Table 1:** Zikavirus *pr*ME pseudotyped HIV-1 used in the study.

Pseudotype	*pr*ME Origin	Genbank	RNA from	*pr*ME Expression Plasmid
Z1-HIV*luc*	Dakar41519	HQ234501	IPC ^a^	pME-Z1
Z2-HIV*luc*	Dakar41524	KX601166	IPC	pME-Z2
Z3-HIV*luc*	ArD158084	KF38119	IPC	pME-Z3
Z4-HIV*luc*	H/PF/2013	KJ776791	EVAg ^b^	pME-Z4

^a^ Institut Pasteur in Cambodia; ^b^ Unit des Virus Emergents, Marseille, France organized by European Virus Archive—GLOBAL.

## Abbreviations

AIDS	acquired immune deficiency syndrome
CRISPR/Cas9	clustered regularly interspaced short palindromic repeats associated protein 9
DMEM	Dulbecco’s modified eagle’s medium
DTT	dithiothreitol
EDTA	ethylenediaminetetraacetic acid
ELISA	enzyme-linked immuno sorbent assay
FCS	fetal calf serum
HIV	human immunodeficiency virus
MERS-CoV	middle east respiratory syndrome coronavirus
PBS	phosphate buffered saline
PBST	PBS including Tween 20
PBSTM	PBST including low-fat milk
SARS-CoV	severe acute respiratory syndrome coronavirus
VSV-G	vesicular stomatitis virus glycoprotein G
